# Impact of a Prospective Simulation-Based Mastery Learning With Deliberate Practice Intervention on Neonatal Intubation

**DOI:** 10.7759/cureus.87239

**Published:** 2025-07-03

**Authors:** Arika G Gupta, Shamik B Trivedi, Shawn M Smith, Mary E McBride, Mark D Adler

**Affiliations:** 1 Division of Neonatology, Department of Pediatrics, Northwestern University Feinberg School of Medicine, Chicago, USA; 2 Division of Hospital Medicine, Department of Pediatrics, Northwestern University Feinberg School of Medicine, Chicago, USA; 3 Division of Cardiology, Department of Pediatrics, Northwestern University Feinberg School of Medicine, Chicago, USA; 4 Division of Emergency Medicine, Department of Pediatrics, Northwestern University Feinberg School of Medicine, Chicago, USA

**Keywords:** deliberate practice, medical education, neonatal intubation, neonatal-perinatal medicine, neonatology, simulation-based education, simulation-based mastery learning

## Abstract

Objective: To design and implement a simulation-based mastery learning (SBML) curriculum utilizing deliberate practice (DP) for neonatal intubation in the delivery room setting. We sought to investigate the impact this curriculum would have on performance and skill retention.

Methods: A prospective single-group SBML with DP intervention to improve intubation success was implemented from 2019 to 2022. Pediatric hospitalists (PHs) and neonatal nurse practitioners (NNPs) who provide care in the delivery room were eligible to participate. An 11-item checklist was developed, and a minimum passing standard (MPS) was set using the Mastery Angoff method. Participants underwent a baseline assessment, followed by DP with expert feedback. Post-intervention assessments were completed until the MPS was achieved, with additional practice rounds if needed. After three to six months, all participants underwent a retention assessment. If MPS was not achieved at retention, further DP was provided until MPS was met.

Results: At the time of enrollment, 95% of participants had limited experience with attempted neonatal intubation (less than five performed), and 14% had never attempted an actual neonatal intubation. At baseline assessment, 95% of participants did not meet the MPS. Participants’ mean scores significantly improved on the intubation checklist from 74% to 99% from baseline to post-training assessment. The intervention was significant and impactful, with effect sizes of >2. At the three-to-six-month retention assessment, 66% of participants met the MPS on the first attempt. Overall, 92% reported an increased confidence level with the procedure and an improvement in their clinical practice.

Conclusions: Our SBML with DP neonatal intubation curriculum is an effective tool to train providers in neonatal intubation for the delivery room environment. As clinical opportunities to learn this procedure continue to decline, programs must develop ways to optimize proficiency in this skill.

## Introduction

The successful transition of a newborn from the intrauterine to the extrauterine environment is dependent on several significant physiologic changes that must occur at the time of birth. While most neonates transition successfully on their own, it is estimated that 10% of neonates require some degree of resuscitation at birth to begin breathing and that approximately 1% require extensive resuscitative measures [1-4].

To be adequately prepared, a team of individuals trained in neonatal resuscitation and airway management should be available to promptly respond and provide resuscitation at all birthing institutions. Achieving effective ventilation is of utmost importance in the successful resuscitation of neonates [4]. The inability to intubate a neonate efficiently and successfully is associated with greater morbidity and mortality. It is paramount that all providers who attend neonatal deliveries be proficient in bag-valve-mask ventilation as well as neonatal intubation [4-6].

Pediatric residents have commonly been the front-line providers attending neonatal deliveries during their newborn nursery or neonatal intensive care unit (NICU) rotations. In the United States, however, this staffing practice at academic teaching hospitals has shifted over time due to a host of factors, including changes in work-hour regulations and requirements for pediatric trainees by the Accreditation Council for Graduate Medical Education (ACGME) [7]. It is now quite common for pediatric hospitalists (PHs) and neonatal nurse practitioners (NNPs) to be front-line providers at term and late-preterm neonatal resuscitations, both at non-academic and academic hospitals [8]. In these situations, PHs and NNPs are expected to provide initial neonatal resuscitative measures in the delivery room, including bag-valve-mask ventilation, intubation, and emergent umbilical line placement, if necessary [8,9]. At many birthing institutions, a neonatologist is not initially present for term or late-preterm deliveries, unless there are anticipated perinatal risk factors to justify their presence. 

At present, there is no uniform standard for teaching neonatal intubation to ensure knowledge and skill acquisition to a level of proficiency for neonatal front-line providers. Numerous studies have demonstrated that neonatal intubation success rates are suboptimal among front-line providers [6,10-12]. This problem is compounded by the increasingly limited opportunities for trainees to practice this skill [13,14]. Thus, early-career neonatal front-line providers, including PHs and NNPs, are likely to be insufficiently prepared to perform this skill when necessary [15].

Simulation-based educational interventions can augment clinical experience and improve clinical performance. Simulation-based mastery learning (SBML) is a specific model of simulation-based education associated with meaningful improvements in educational and clinical outcomes [16,17]. In SBML interventions, learners acquire knowledge and skills through deliberate practice (DP) with feedback until they have met a pre-determined minimum passing standard (MPS), which is set following an established methodology [17-20]. Learning is not time-fixed, and learners continue to practice with ongoing directed feedback until they can achieve the MPS [17,20]. While there is published work on the use of SBML interventions in other pediatric procedures [21-24], to our knowledge, there is no published literature studying the impact of SBML with DP on neonatal intubation skill acquisition and retention. In this work, we describe the design and implementation of our SBML neonatal intubation intervention.

## Materials and methods

Study design

We conducted a prospective pretest-posttest, single-arm SBML intervention to improve neonatal providers’ intubation performance in a simulated setting.

Setting

A single urban academic tertiary children’s hospital from December 2019 to January 2022. Participants were recruited in two groups - Cohort 1 and Cohort 2, before and after the COVID-19 pandemic, which precluded in-person training for a while.

Participants

At our institution, PHs and NNPs often provide front-line care to neonatal patients admitted to the NICU. In this role, they also routinely attend term and late-preterm deliveries independently. All early-career PHs and NNPs who practice in the NICU, defined as having less than two years of experience in their current role, were eligible to participate in our study. We intentionally chose to enroll early-career providers, as they were most likely to benefit from this intervention. Eligible providers were recruited via email and invited to participate voluntarily. Our institution’s institutional review board determined this study was exempt. Verbal consent was obtained at the time of enrollment. 

Intervention

Upon enrollment, all participants completed an anonymous survey to provide baseline information regarding years of experience in their role and their level of clinical experience and confidence with neonatal airway management.

Participants were individually brought into the simulation lab, which was set up to mimic a neonatal resuscitation bay in the delivery room, and orientation was provided to the room and the simulator. The neonatal resuscitation bay in the simulation lab was equipped with a radiant warmer, patient monitor, and all necessary equipment for a neonatal delivery and intubation. Subsequently, each participant individually underwent a baseline assessment (pre-test) of their neonatal intubation performance using an 11-item intubation checklist. We used SimNewB (Laerdal, Wappingers Falls, NY), a realistic newborn simulator designed for neonatal resuscitation training and advanced airway management. This simulator has been studied to have high physical and functional fidelity among a variety of neonatal airway simulators [25]. A patient monitor with vital signs mimicking those typically available in the delivery room environment was provided during the scenario. The case scenario for the pre-test involved an apneic and bradycardic term newborn in the delivery room, who responded to initial mask positive-pressure ventilation (PPV) and required intubation for persistent apnea. The same case scenario was used at all visits, and participants were explicitly asked not to share details of the case with other participants. Immediately after the pre-test was completed, each participant received the study intervention, which was composed of one-on-one individualized training with directed expert feedback provided by the study author (AGG). The content of the training focused on performance gaps identified by each individual’s performance during the pre-test. While the checklist items were used as a guide to direct these DP sessions, participants were also encouraged to bring up any additional topics of inquiry regarding the procedure. All participants received the intervention, DP with expert feedback, until they demonstrated the ability to achieve the MPS. The checklist items were not disclosed to the learners.

Each participant was then asked to return within one to two weeks for a post-training assessment (post-test), during which the same simulation equipment, scenario, and intubation checklist were utilized. If the participant did not achieve the MPS on the checklist at the time of this assessment, they underwent further one-on-one DP time and directed expert feedback until the participant demonstrated the ability to achieve the MPS. If further DP was required, participants returned one to two weeks later for a second post-training assessment (second post-test).

Once an individual met the MPS at a post-training assessment or second post-training assessment, they were asked to return for a retention assessment approximately three to six months later. At this retention visit, they underwent a reassessment of neonatal intubation performance, using the same simulation equipment, scenario, and intubation checklist. If the participant did not achieve the MPS, they received additional DP and were asked to return in one to two weeks for a second retention assessment. During all visits, participants were allowed as much time as they needed for DP and directed expert feedback until they demonstrated successful performance of each checklist item.

Instrument development

We developed an 11-item intubation checklist adapted from the International Network for Simulation-based Pediatric Innovation, Research, and Education (INSPIRE) neonatal intubation competency assessment tool [26]. The INSPIRE tool was developed primarily for use with simulated or clinical elective neonatal intubations, rather than emergent intubations in the simulated delivery room environment. Thus, we adapted this checklist based on a review of relevant literature, best practices, and expert opinion to meet the needs of our educational setting and intervention. Following this work, a group of five board-certified neonatology attending physicians from our academic institution, with experience performing neonatal intubation, participated in a modified Delphi process [27]. Each member of the expert panel was asked to independently assess each item on the checklist and invited to revise any items, as necessary. The initial checklist was then modified based on the collated feedback from experts. The updated checklist was then returned to the same group of experts for additional review. Any additional changes that were recommended by the second review were then incorporated if there were no contradictory suggestions provided by other reviewers. Once we achieved consensus amongst the expert panel members, the checklist was then pilot tested with non-study subjects in the simulation lab to ensure usability. The checklist was subsequently modified based on the results of pilot testing to improve the clarity of checklist items. Ultimately, our assessment tool was an 11-item dichotomous response (“correct” or “incorrect/not done”) checklist, specifically designed for the assessment of emergent neonatal intubation in the delivery room environment. All checklist items were equally weighted. Key members of the expert panel were then asked to review the checklist again to ensure a final consensus on the items. Subsequently, a new panel of experts, composed of 10 academic neonatologists, two PHs, and two pediatric emergency medicine physicians, participated in the MPS setting process following the Mastery Angoff methodology [18]. All panelists were from a single academic institution and varied in gender and years of clinical experience. (See Table 2 in the Appendix for the final version of the intubation checklist.) The Mastery Angoff standard-setting process resulted in an MPS set at 100% on the intubation checklist. Of note, it is not unusual with this method of standard setting to result in an MPS at >90%.

Checklist raters (AGG and SBT) completed two hours of rater training on the checklist. All performance assessments were graded by one unblinded instructor (AGG) in real time, and all baseline assessments (pre-tests) were video recorded to be reviewed by a second author (SBT). A random sampling of baseline assessments was analyzed for interrater reliability (IRR). We compared baseline and post-training assessment checklist scores to measure the impact of the intervention. All participants from Cohort 2 of the study completed an anonymous electronic course evaluation questionnaire, about 9-15 months after study completion. Participants rated their overall satisfaction with the curriculum, relevance to their clinical practice, and change in confidence in neonatal intubation in clinical practice after participation in the study. 

Statistical analysis

Descriptive statistics regarding participants’ characteristics, self-reported experience, and level of confidence with neonatal intubation were collected using an anonymous electronic questionnaire. Data collected included provider type (PHs vs. NNPs), years of clinical experience in current role, prior neonatal intubation experience, and comfort level with the procedure.

We report individual participants’ checklist scores, comparing pre-test and post-test scores, to describe the impact of the intervention. We also compare individual participants’ scores on the post-test and retention assessment to describe participant retention after the intervention. We used two-tailed paired t-tests with a significance level set at p<0.05. We also report the proportion of participants who met the MPS at each assessment period. Effect sizes are reported as Cohen’s d coefficients, comparing baseline assessment to post-training assessment, and baseline assessment to final (mastery) assessment. We evaluated checklist score inter-rater reliability for the neonatal intubation procedure using Gwet’s AC1 coefficient [28]. Statistical analyses were performed using STATA 18.0 (StataCorp, 2023. Stata Statistical Software: Release 18.0. College Station, TX: StataCorp LLC).

## Results

**Figure 1:**
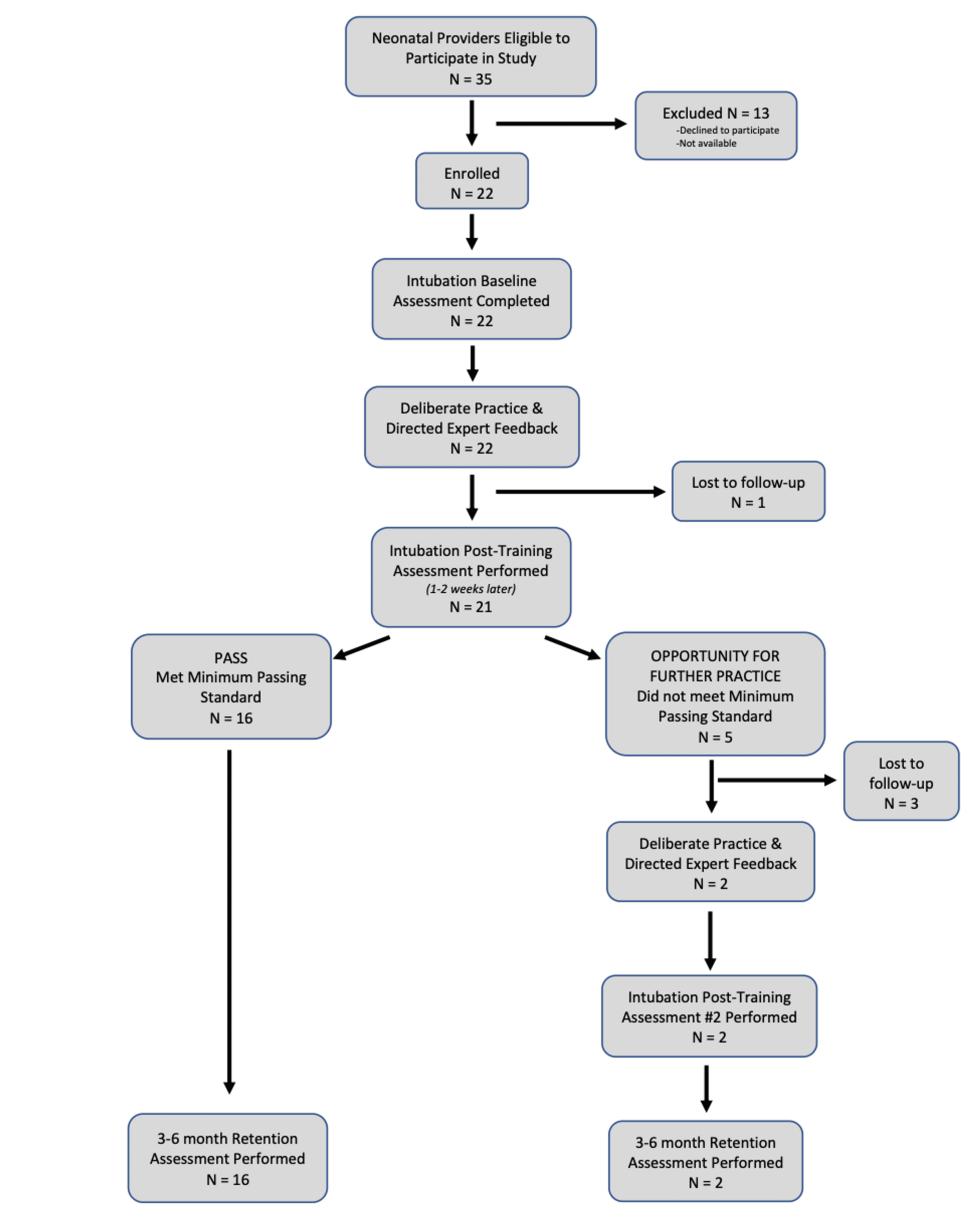
Flow diagram illustrating the recruitment, timeline, and retention of study participants

A total of 35 neonatal providers were eligible to participate in the study during the study period. A total of 22 neonatal providers (63%) were enrolled in the study protocol (including both Cohorts 1 and 2). Four providers did not complete the study protocol due to illness or scheduling barriers. Thus, 82% of enrolled participants completed the entire study protocol. Figure 1 summarizes recruitment, timeline of visits, and retention of participants in the study protocol. Table 1 displays participants’ characteristics and self-reported experience with neonatal intubation. Of the enrolled participants, 64% (n=14) were PHs, and 36% (n=8) were NNPs. The majority (68%) of participants had less than one year of clinical experience in their current role at the time of enrollment. Most of the participants (95%) had limited clinical experience with attempted neonatal intubation (less than five performed), and 14% had never attempted a neonatal intubation. Thirty-two percent had never successfully intubated an actual neonate.

**Table 1:** Participant characteristics at the time of enrollment into the study

Characteristics	Number of Participants (%)
		Clinical Role	
Pediatric Hospitalist	14 (64%)
NNP	8 (36%)
Years of Clinical Experience	
		< 1 year	15 (68%)
1-2 years	7 (32%)
Number of Actual Neonatal Intubations Performed Prior to Participation in Study	Number of Participants (%)
Attempted Intubations	
0	3 (14%)
1-5	18 (82%)
>5	1 (5%)
Successful Intubations	
0	7 (32%)
1-5	14 (64%)
>5	1 (5%)

**Figure 2:**
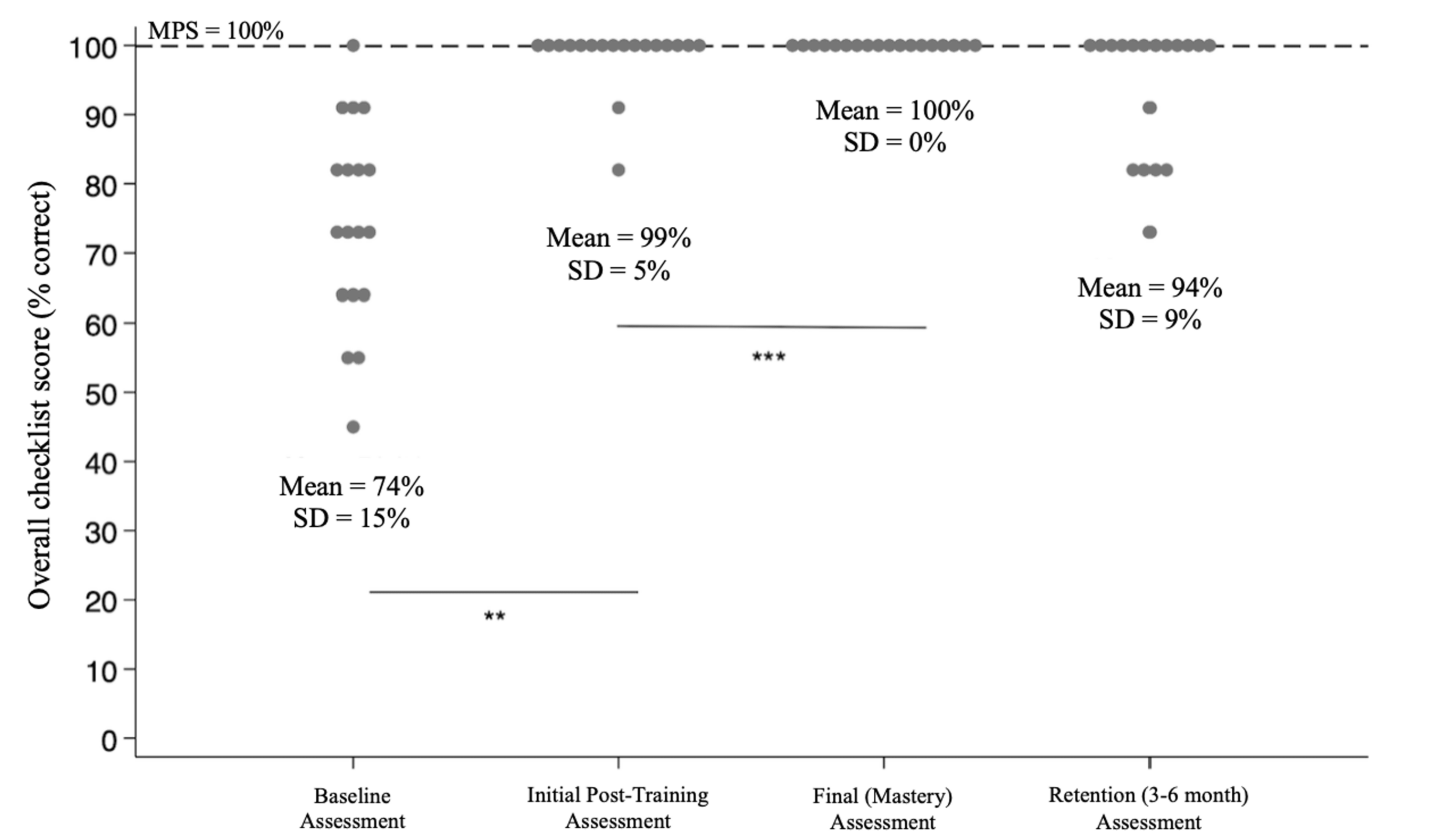
SBML-trained neonatal provider baseline assessment, initial post-training assessment, and final (mastery) assessment on an 11-item intubation checklist Each circle on the graph represents an individual neonatal provider. SBML = Simulation-based mastery learning; MPS = minimum passing standard; SD = standard deviation. ** and *** indicate a significant difference between scores at p<0.05

Ninety-five percent of participants did not meet the MPS at baseline assessment. The participants significantly improved their performance on the intubation checklist from a mean score of 74% (SD=15%) at baseline assessment to 99% (SD=5%) at the initial post-training assessment, and all achieved mastery (MPS) at the final assessment (Figure 2). The intervention was significant and impactful, with effect sizes of >2. Five (24%) of the 21 participants required more than one attempt at the post-training assessment to meet the MPS. These five participants required an average of 13 minutes of additional DP time to meet the MPS after their first post-training assessment. The most frequently missed items on the checklist at post-training assessment were 1) checking for an available stethoscope during preparation for the procedure and 2) pivoting or rocking the laryngoscope handle during the procedure. 

At the three to six-month retention assessment, the mean score for all participants was 94% (SD=9%), with 67% of them meeting the MPS at the first attempt and the remainder requiring additional deliberate practice to meet the MPS at a subsequent retention assessment. The participants who required additional DP required an average of 18 minutes of additional time to meet the MPS after their first retention assessment. All participants who completed their retention assessment less than five months after their initial post-training assessment met the MPS on the first attempt. For those who completed their retention assessment greater than or equal to five months later, 54% of these participants met the MPS on their first retention assessment.

Seventeen baseline assessments were dual-rated to assess IRR, with the second rater utilizing video recordings of assessments to complete the checklist. The average Gwet AC1 coefficient for our instrument was 0.58, with three items with substantial variation in agreement across items (three items with coefficients <0.3).

Of the participants who were recruited during Cohort 2 of the study, 100% of the participants (n=11) completed a course evaluation questionnaire, which was sent out approximately nine to 15 months after participation was complete. Of these participants, 100% agreed that the simulation curriculum was relevant and useful to their clinical practice. In addition, 91% of participants found that their participation in the curriculum has helped to improve their clinical practice. Finally, 91% reported an increase in confidence level in performing neonatal intubation after participation in this study.

## Discussion

We have designed and implemented an SBML with DP neonatal intubation curriculum for early-career neonatal providers. This program was feasible to implement and had a strong educational impact in that all participants were able to achieve performance at the mastery level. This curriculum was well-received by the learners, and they found the content to be useful and relevant to their clinical practice. In addition, participants reported that involvement in this curriculum increased their confidence level in actual clinical care. Despite having completed rigorous training, unit-specific orientation, and onboarding shifts, the early-career providers enrolled in this study reported low baseline confidence levels in neonatal intubation. Furthermore, they reported a low volume of clinical experience in neonatal intubation and demonstrated low baseline performance in simulated neonatal intubation. Nevertheless, these same clinicians are expected to be front-line providers in the delivery room and are expected to provide immediate, effective ventilation and intubation when necessary. Thus, there is a clear need for this curriculum within this provider population.

In this study, we observed a significant improvement in simulated neonatal intubation performance from the initial baseline assessment to the final mastery assessment. However, skill retention at three to six months after mastery assessment was found to be suboptimal. Consistent with findings from previous research [21,24], our data demonstrated a decline in skill retention over time following initial mastery achievement. Participants whose retention was assessed five months or more after achieving mastery were more likely to require additional DP to regain mastery. Notably, the average DP time required to reattain mastery following the retention assessment was shorter than the DP time required to achieve initial mastery. These findings support the need for regular DP refreshers, which may be more time-efficient than initial training, to maintain performance standards.

Historically, the ACGME Program Requirements for Graduate Medical Education (GME) in pediatrics have included competence in neonatal intubation as an expected outcome for graduating residents [7]. However, this requirement has recently been removed, and the mandated number of NICU rotations has also been reduced [7]. These changes reflect a broader educational shift toward preparing residents for general pediatric practice. As a result, the decline in NICU exposure and procedural training in the US is widening the gap in clinical experience, leaving many graduating pediatric residents underprepared for neonatal airway management, including intubation. This gap also extends to neonatal-perinatal medicine fellowship trainees, who will be entering their training with limited prior experience. These trends also have significant implications for NNPs, who are expected to develop these critical skills during their training and onboarding. A curriculum focused on neonatal intubation is, therefore, well suited for graduating pediatric residents and student NNPs preparing for frontline roles in neonatal deliveries.

This study has several limitations. First, our study was conducted at a single academic center. In addition, we intentionally limited this intervention to the delivery room, as this is most commonly the environment in which neonatal front-line providers are expected to independently perform emergent intubation. Thus, our results cannot be extrapolated to other settings, such as non-delivery room and/or non-emergent neonatal intubations. Second, we had a limited number of participants enrolled in this study, as it was optional to participate, and we aimed to study the impact of this curriculum only on early-career neonatal front-line providers. Therefore, we were limited in the number of eligible participants at our institution. Third, our two raters were not fully blinded during this study, as it was not feasible to blind raters completely as to the identity of each participant. However, the second rater exclusively watched video recordings of each participant’s performance, with each video file name being de-identified, and the participant’s face was intentionally not captured. The second rater’s scoring was significantly impacted by the limitations of the video recordings, as the intubation checklist was not initially designed to be suitable for rating via video review. However, due to COVID-19 pandemic restrictions and concerns, the second rater relied primarily on video review. For the three checklist items with substantial variation in agreement, the ability to see certain behaviors on video recordings was felt to be a possible source of disagreement. This impacted our ability to assess interrater reliability agreement significantly.

This study demonstrates the feasibility, effectiveness, and educational impact of an SBML intervention with DP for training frontline neonatal providers to perform neonatal intubation at a mastery level. The curriculum was successfully implemented at a high-volume academic children’s hospital. Further research is needed to evaluate its impact on clinical performance.

## Conclusions

We demonstrated that an SBML curriculum with DP was effective for training novice providers to the mastery level in neonatal intubation. Current training paradigms are insufficient in preparing front-line providers for this necessary life-saving skill. In neonatal-perinatal medicine, we must implement efficient and effective strategies to ensure that our providers caring for vulnerable newborns in the delivery room are optimally prepared to do so. Future work in this area should address the impact that such interventions have on clinical outcomes.

## Appendices

**Table 2:** Simulation-based mastery learning (SBML) neonatal intubation checklist for the delivery room

Simulation-Based Mastery Learning (SBML) Neonatal Intubation Checklist for the Delivery Room		
Participant ID: ☐ ☐ ☐ ☐	Evaluator: ____________________________	
				Done Correctly	Done Incorrectly or
	Not Done			
Preparation: (Confederate reads 'script' to participant, prior to starting procedure)		
			Checks all of the following equipment, prior to starting procedure: stylet and stylet position (if using stylet), range of appropriate size endotracheal (ET) tubes, laryngoscope handle and appropriate size Miller blade (1 or 0 blade acceptable) (for SBML training, Confederate states "Is there anything else you need me to get ready for you?")	☐	☐
Checks all of the following equipment, prior to starting procedure: suction, CO_2_ detector, stethoscope	☐	☐
Checks all of the following equipment, prior to starting procedure: bag and mask or T-piece resuscitator with mask, O_2_ flow and blender	☐	☐
Selects appropriately sized ET tube to start with for procedure (based on either the patient's estimated weight or gestational age)	☐	☐
Appropriately positions airway to optimize view (learner must perform at least one of these: position bed height, adjust head position, utilize shoulder or neck roll) (for SBML training - if not done appropriately, Confederate should ask "Do you need anything from me before starting the procedure?")	☐	☐
Procedure				
					Inserts the laryngoscope blade into the mouth using the left hand	☐	☐
Demonstrates appropriate technique to lift the handle of the laryngoscope forward (does not pivot/rock the handle)	☐	☐
Demonstrates appropriate use of suction (for SBML training, Confederate will state "The baby has a lot of secretions.")	☐	☐
Demonstrates appropriate insertion of ET tube (i.e., using right hand to insert, does not obscure view while inserting, and inserts/adjusts ET tube to approximately correct depth)	☐	☐
Employs appropriate techniques to confirm ET tube placement (confirms all three are done): auscultation of bilateral breath sounds, bilateral chest rise, and CO_2_ detector color change (for SBML training, Confederate will state "Can you tell me what the measurement at the lip is?")	☐	☐
Ensures the ET is secured appropriately - for SBML training, confederate will secure the tube loosely and ask "Are you happy with how this taping looks?", providing the learner with the opportunity to request that it be re-secured	☐	☐
